# *Corynebacterium bovis* Surgical Site Infection and Abscess Formation: A Case Report

**DOI:** 10.1002/ccr3.71413

**Published:** 2025-11-14

**Authors:** Grace VanGorder, Emily Peterson

**Affiliations:** ^1^ Penn State College of Medicine Hershey Pennsylvania USA; ^2^ Department of Plastic Surgery Mount Nittany Health State College Pennsylvania USA

**Keywords:** abscess, blepharoplasty, case report, *Corynebacterium bovis*, plastic surgery, surgical site infection

## Abstract

*Corynebacterium bovis* is primarily known for causing mastitis in cows. *C. bovis* infection in humans is quite rare with only 11 cases reported in the United States and 26 globally. A 69‐year‐old female presented with a left lower eyelid abscess following removal of a retained suture after bilateral upper and lower blepharoplasty. The abscess was drained, and bacterial culture identified *C. bovis* . The patient was treated with oral doxycycline with complete resolution of the infection. No intraorbital, intracranial or blood infections resulted from the superficial infection. The authors of this report hope to provide guidance to clinicians on treatment options for successful eradication of human soft tissue infection caused by this bacterium.

**Summary**

- Human infection with *Corynebacterium bovis* , though uncommon, has been reported with increasing frequency in recent years.
- In the event of soft tissue infection caused by *C. bovis* , incision and drainage followed by oral doxycycline therapy can be an appropriate and effective treatment option.

## 1 Introduction

*Corynebacterium bovis* is a gram‐positive, catalase‐positive, club‐shaped bacteria most notable for its role in infection of the udder of a cow resulting in mastitis and decreased milk production. The corynebacteriaceae family also includes *C. diphtheriae*, which causes diphtheria, an illness well known to the human host [1]. In the past century, only 26 cases of *C. bovis* have been reported in humans. Infection of surgical site is the most common mode of infection by this bacterium (Table 1). The most common mode of infection by this bacterium is through surgical site contamination (Table 1). Infection can occur via two primary mechanisms: breaches in sterile technique during surgery or inadequate postoperative wound management, where unsanitary conditions expose the open wound to the bacterium.

**TABLE 1:** Documented *Corynebacterium bovis* infections.

Infection	Source cultured	Treatment course	Country	Publication (year)
Ventriculojugular shunt infection, glomerulonephritis	Blood culture	IV penicillin, followed by oral erythromycin and rifampin	USA	Bolton et al. (1975) [6]
Meningitis	CSF	Ampicillin followed by penicillin, streptomycin, and chloramphenicol	England	Vale and Scott (1977) [1]
	CSF	Cloxacillin		
Spinal epidural abscess/meningitis				
	Wound exudate	Ampicillin		
Leg ulcer				
	Aural drainage	Neomycin/Betamethasone drops, followed by oral ampicillin		
Chronic otitis media				
	Blood	Cephaloridine, erythromycin, and fusidic acid		
Subacute endocarditis				
	Blood	Erythromycin and fusidic acid		
Subacute endocarditis				
	Conjunctival fluid	Bacitracin/Polymyxin B ointment	Switzerland	Duty et al. (2004) [7]
Purulent conjunctivitis				
	Blood	IV vancomycin, meropenem, tigecycline, and polymyxin, followed by IV vancomycin	USA	Dalal et al. (2008) [8]
Line‐related septicemia				
	Sonication fluid	IV imipenem, followed by oral amoxicillin	Switzerland	Achermann et al. (2009) [2]
Prosthetic joint infection				
	Pus	Oral trimethoprim‐sulfamethoxazole (TMP/SMX)	Philippines	Vargas et al. (2009) [9]
Brain abscess				
	Blood	IV amikacin and teicoplanin	Morocco	Sbaai et al. (2011) [10]
Septicemia				
	Conjunctival fluid	Tobramycin drops and oral amoxicillin/clavulanic acid	USA	Chow et al. (2013) [11]
Purulent conjunctivitis				
	Conjunctival fluid	Ofloxacin drops		
Purulent conjunctivitis				
	Conjunctival fluid	Erythromycin ointment and oral TMP/SMX		
Purulent conjunctivitis				
	Wound exudate	Oral doxycycline		
Skin ulcer				
(5) Wound (unspecified)	Wound exudate	Unknown	USA	Cheleuitte‐Nieves, Christopher et al. (2018) [14]
Preseptal cellulitis	Conjunctival fluid	IV amoxicillin/clavulanic acid, followed by oral amoxicillin/clavulanic acid	Netherlands	Meeuwes et Wolfs (2019) [12]
Corneal abscess	Conjunctival fluid	Gentamicin drops, vancomycin drops, and oral ciprofloxacin	England	Elsheikh et al. (2021) [13]
Injection port site breast abscess	Pus	IV imipenem	China	You et al. (2023) [3]
Superficial and deep surgical site infection of scalp	Wound exudate	IV ceftriaxone and oral metronidazole	Israel	Gabay et. al (2023) [4]
Granulomatous cystitis	Cyst fluid	PO Doxycycline	Belgium	Lebbe et. al (2024) [5]

No current established treatment protocol exists for *C. bovis* infection due to its rarity in humans. However, *C. bovis* is susceptible to tetracycline, enrofloxacin, ampicillin, rifampicin, and ciprofloxacin in the laboratory setting [3] and resistant to clindamycin and benzylpenicillin [4]. The authors of this report hope to provide guidance to clinicians in treatment decision‐making processes for successful eradication of this bacteria in cases of human soft tissue infection.

## 2 Case History and Examination

A 69‐year‐old female was evaluated for a secondary bilateral upper and primary lower blepharoplasty. Her medical history includes Ehlers–Danlos syndrome, hypermobile type, mitral regurgitation, asthma, migraine, arthritis, and osteoporosis. Past surgical history included a bilateral upper blepharoplasty 5 years prior and facial rhytidectomy 6 months prior.

The blepharoplasties were performed in a hospital affiliated ambulatory surgery center under general anesthesia. The upper blepharoplasties were performed first by removing excess upper eyelid skin and excess fat from the medial compartments. A transcutaneous approach was utilized for the lower blepharoplasties. A skin‐muscle flap was raised, fat was resected, and a conservative 4 mm strip of skin was removed. A canthopexy was performed using a 5‐0 PDS suture. All incisions were closed using simple interrupted 6‐0 nylon sutures. Bacitracin ophthalmic ointment was applied to the incisions. There were no intraoperative complications.

The patient was discharged from the ambulatory surgery center with a prescription for bacitracin ophthalmic ointment to be used three times daily for 7 days. She was seen in routine follow‐up postoperatively, with anticipated mild bruising and edema, postoperative day 7 for suture removal. She self‐discontinued antibiotic ointment postoperatively. On postoperative day 7, upper and lower eyelid sutures were removed by the office nurse without complication. There was no evidence of infection or incisional separation at this time. She returned postoperative day for follow‐up by the attending surgeon with expected postoperative findings.

Six weeks postoperatively, the patient was seen for routine follow‐up. A small piece of retained nylon suture was noted adjacent to the left lateral canthus, which was removed from her left lower eyelid incision with sterile scissors and forceps. The patient returned 2 days later due to suspicion for additional retained suture material in the left lower lid incision. This was lifted out using sterile scissors and a 30‐gauge needle.

Ten weeks postoperatively, the patient contacted the office due to a several day history of an enlarging bump on the left lateral eyelid at the site of the previously retained suture. She had attempted conservative treatment with warm compresses without improvement. A 1‐cm abscess located at the lateral canthus of the left lower eyelid was observed and managed with an incision and drainage procedure in the office. A culture was obtained, and the wound was irrigated with normal sterile saline solution. The patient was advised to use erythromycin ophthalmic ointment and apply warm compresses.

## 3 Investigation and Differential Diagnosis

Bacterial cultures identified the growth of *C. bovis* . The patient mentioned attending a farm show after the retained suture extraction, which may have been the source of the bacteria. The case was reviewed with an infectious disease colleague, who recommended doxycycline 100 mg orally for 14 days as the most appropriate treatment. A CT scan of the bilateral orbits, both with and without contrast, showed no periorbital abnormalities or abscess. However, it incidentally revealed an enhancing mass in the left falx, consistent with a meningioma. She showed no focal neurological deficits and is stable on neurology follow‐up.

## 4 Conclusion and Results

The patient returned to the office 4 weeks later for another incision and drainage due to a recurrent 3‐mm abscess. Another 10‐day course of doxycycline hyclate 100 mg twice daily was initiated; the wound culture showed no growth, so antibiotic treatment was discontinued. The patient was evaluated 3 weeks later, and the abscess had mostly resolved with only a 1 mm raised, slightly erythematous lesion remaining.

## 5 Discussion

*C. bovis* infection in dairy cattle results in inflammation of the mammary gland, blocked milk ducts, reduced milk production, fever, and lethargy. Symptoms usually manifest 7–10 days after infection. Prevention can be achieved through post‐milking teat dipping and proper milking hygiene. While this bacterium is common and often considered a commensal bacterium in bovine utters, reported opportunistic infections in humans range in severity mild abscesses to severe infections such as meningitis and sepsis.

Currently, there are no established guidelines for the antibiotic treatment of soft tissue *C. bovis* infections in humans. In case of infection, the most effective treatment for cows is penicillin. However, with an increasing rate of beta lactam resistance in humans, this may not be the most appropriate option. Successful treatment for human *C. bovis* infections have been treated with broad‐spectrum antibiotics such as ampicillin, erythromycin, doxycycline, and other combined therapies, as demonstrated in Table 1. Many of these patients were not treated penicillin and had complete resolution of *C. bovis* infection. The literature review indicates that human infection with *C. bovis* is uncommon. However, cases of human infection have been increasing in frequency in the 21st century. While the majority of infected patients were able to recover following antibiotic treatment, there was one case of *C. bovis* infection resulting in endocarditis and subsequent death [1]. Documenting successful treatments of such infections is essential for developing an evidence‐based treatment protocol to guide effective treatment.

This case highlights the need for better patient education on postprocedure wound care. The patient's exposure to farm animals likely led to *C. bovis* infection after suture removal. Alternative transmission modes, such as clinic contamination and inadequate precautions during suture removal, should also be considered.

Human infection with *C. bovis* , though uncommon, has been reported with increasing frequency in recent years. Due to the absence of well‐defined treatment protocols, it is essential to document successfully treated cases to aid in establishment of evidence‐based guidelines for future management of such infections.

As always, it is important to follow guidelines for infection prevention in the workplace by maintaining a clean environment and using proper procedural technique, as well as educate patients on how to care for surgical wounds. In the event of soft tissue infection caused by *C. bovis* , incision and drainage followed by oral doxycycline therapy can be an appropriate and effective treatment option.

## Author Contributions

**Grace VanGorder:** conceptualization, data curation, formal analysis, investigation, methodology, writing – original draft. **Emily Peterson:** conceptualization, data curation, investigation, methodology, project administration, resources, supervision, writing – review and editing.

## Ethics Statement

All authors have agreed to authorship, read and approved the manuscript, and given consent for publication of the manuscript.

## Consent

Written informed consent was obtained from all patients to publish this report in accordance with the journal's patient consent policy.

## Conflicts of Interest

The authors declare no conflicts of interest.

## Data Availability

Data sharing not applicable to this article as no datasets were generated or analyzed during the current study.
